# Supplementary figures and images for: FMNL2 regulates actin for endoplasmic reticulum and mitochondria distribution in oocyte meiosis
Source: eLife. 2024 May 15;12:RP92732. doi: 10.7554/eLife.92732 (PMC11095938; doi:10.7554/eLife.92732)

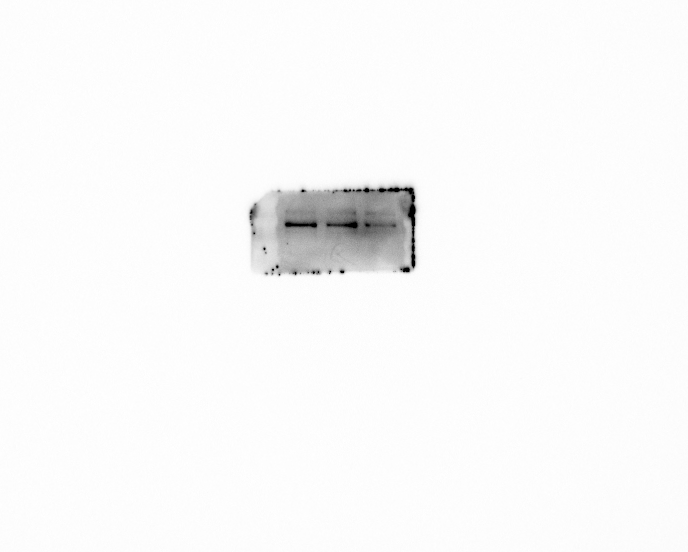

Supplement: Figure 1—source data 1. [file elife-92732-fig1-data1.zip › Fig 1/A---fmnl2---gv-mi-mii.tif]

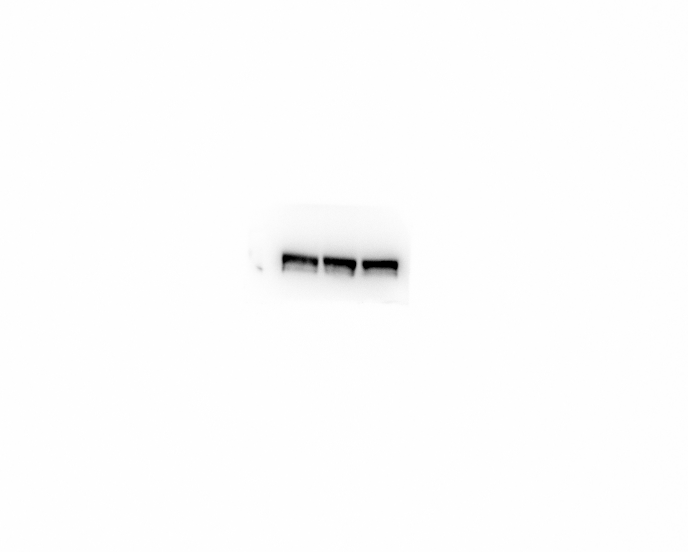

Supplement: Figure 1—source data 1. [file elife-92732-fig1-data1.zip › Fig 1/A---tubulin---gv-mi-mii.tif]

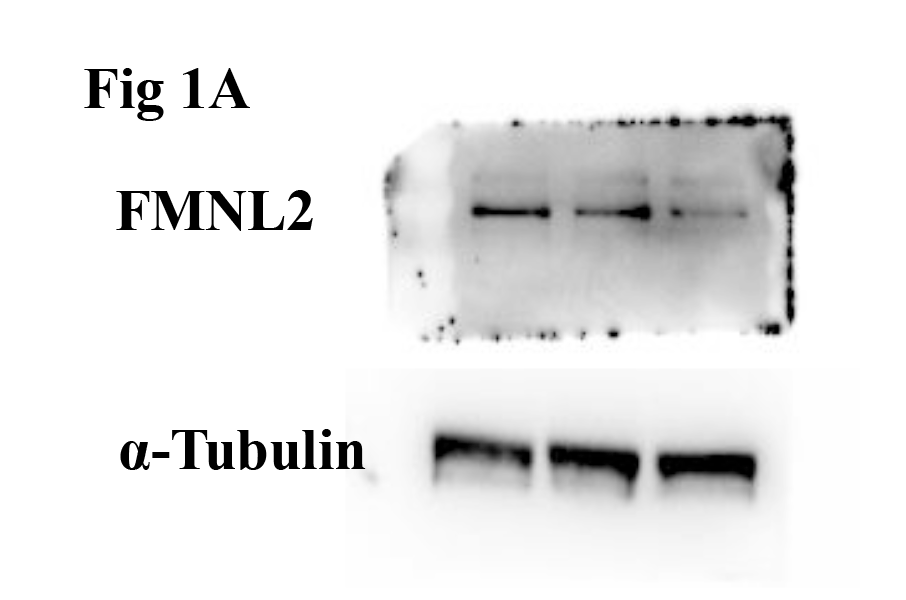

Supplement: Figure 1—source data 2. [file elife-92732-fig1-data2.zip › Figure 1 source data 2.tif]

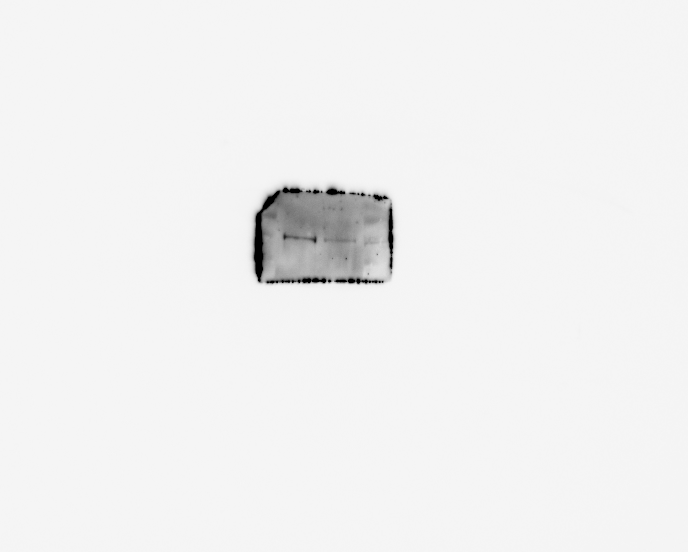

Supplement: Figure 2—source data 1. [file elife-92732-fig2-data1.zip › Fig 2/A---Fmnl2--c-Fmnl2KD.tif]

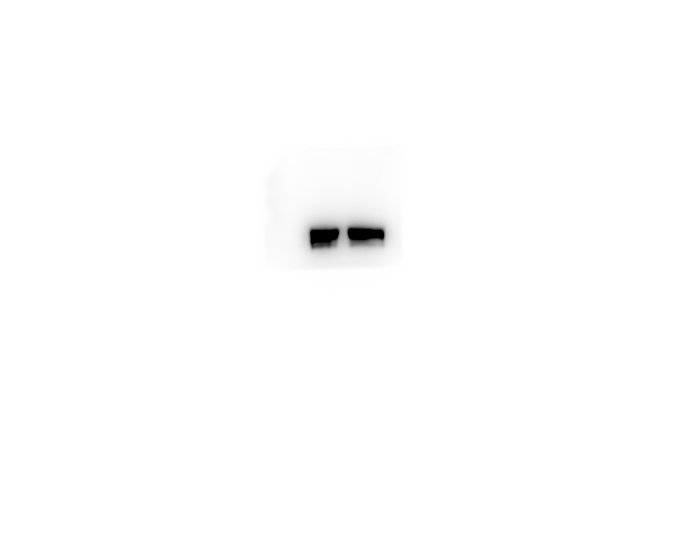

Supplement: Figure 2—source data 1. [file elife-92732-fig2-data1.zip › Fig 2/A---tubulin--c-Fmnl2KD.tif]

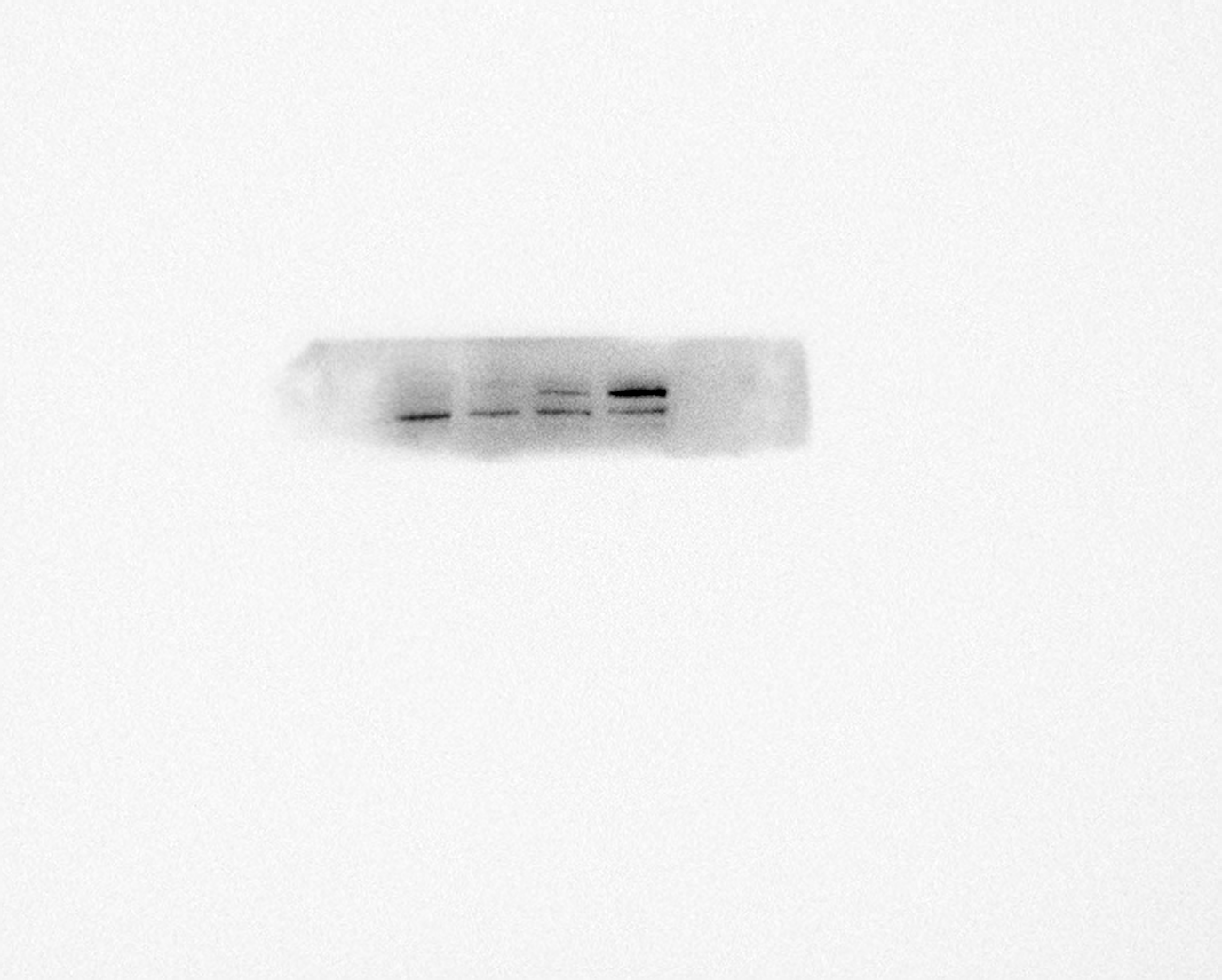

Supplement: Figure 2—source data 1. [file elife-92732-fig2-data1.zip › Fig 2/F---fmnl2---c-KD-200-400.tif]

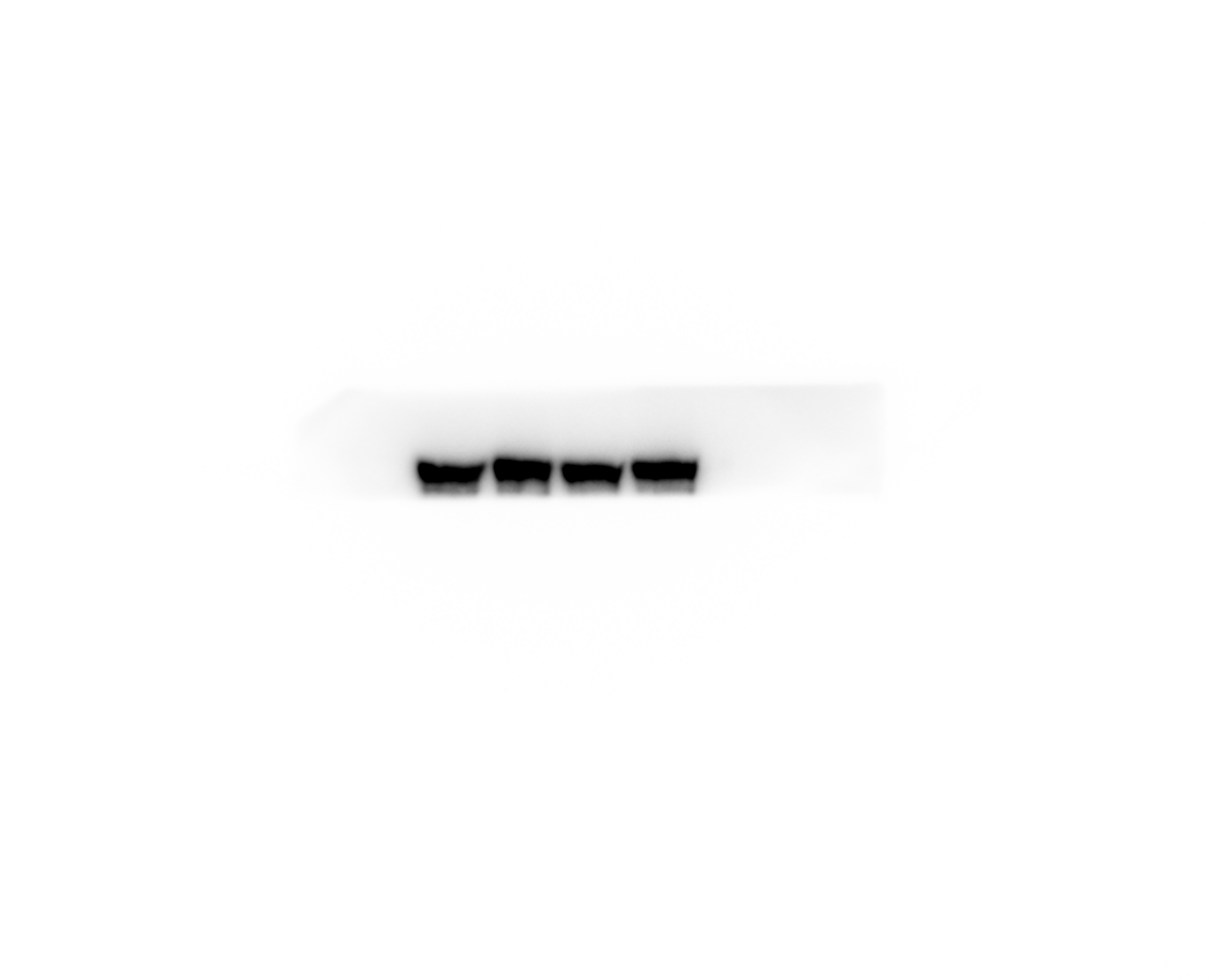

Supplement: Figure 2—source data 1. [file elife-92732-fig2-data1.zip › Fig 2/F---tubulin---c-KD-200-400.tif]

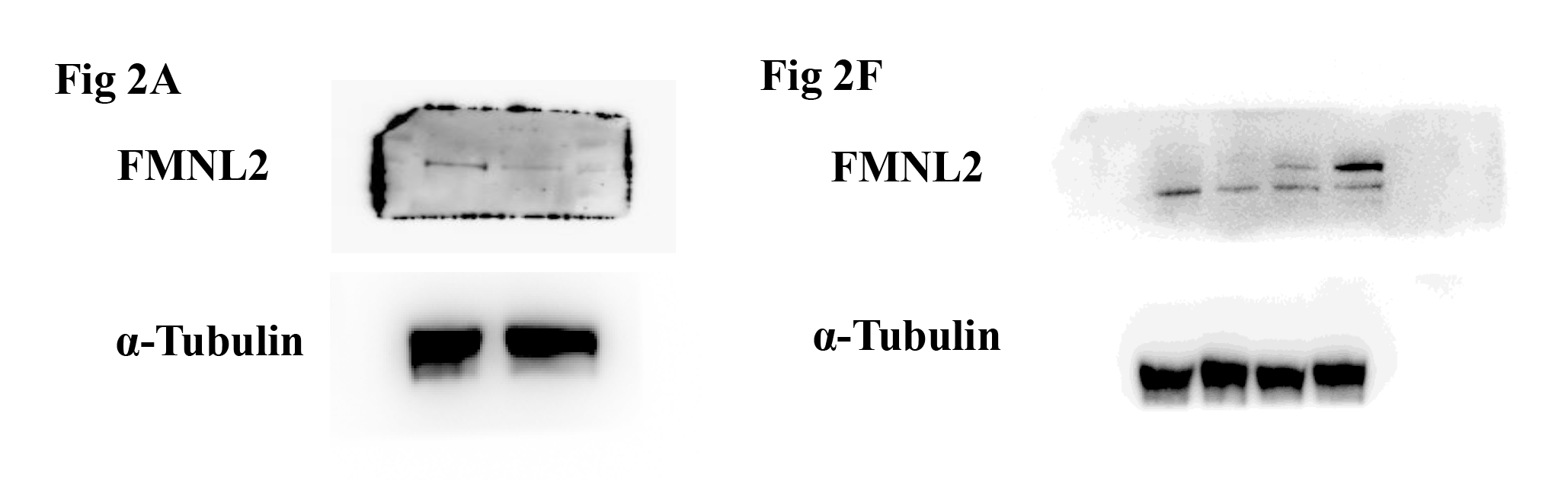

Supplement: Figure 2—source data 2. [file elife-92732-fig2-data2.zip › Figure 2 source data 2.tif]

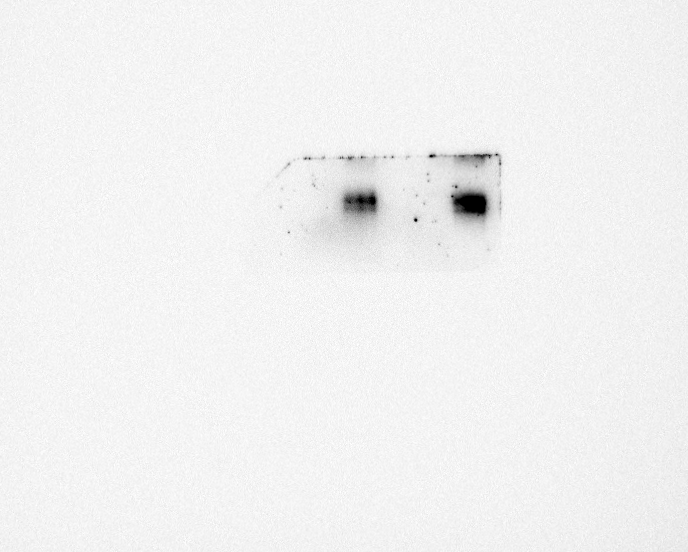

Supplement: Figure 4—source data 1. [file elife-92732-fig4-data1.zip › Fig 4/H---Arp---input-fmnl2-input-fmnl2.tif]

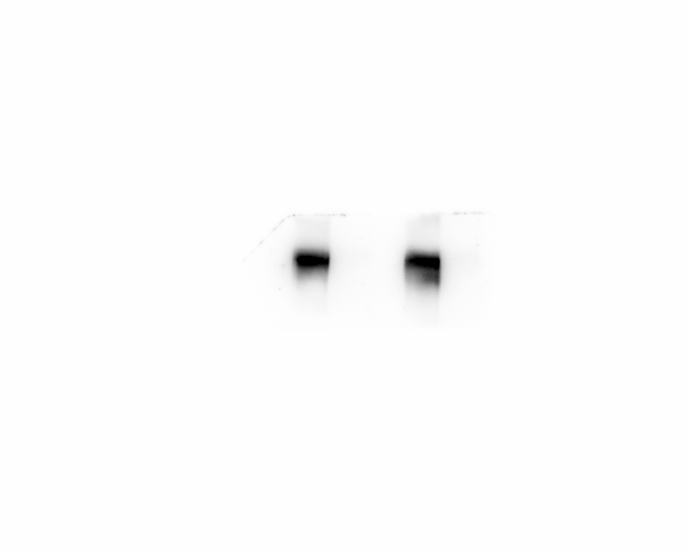

Supplement: Figure 4—source data 1. [file elife-92732-fig4-data1.zip › Fig 4/H---fascin---input-fmnl2-input-fmnl2.tif]

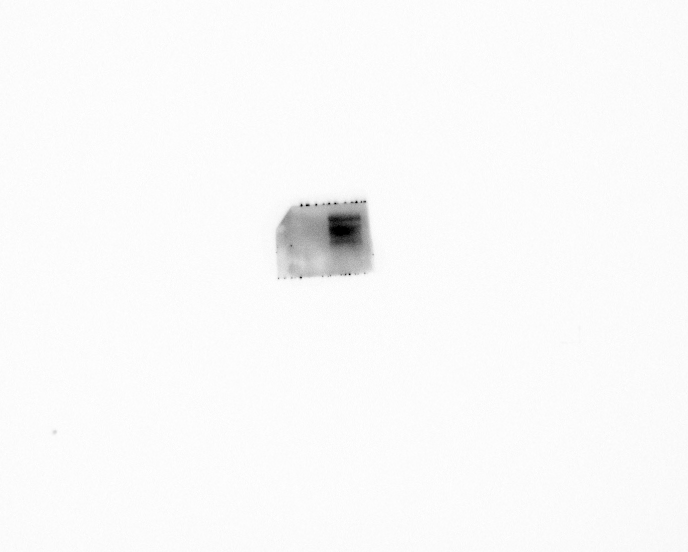

Supplement: Figure 4—source data 1. [file elife-92732-fig4-data1.zip › Fig 4/H---Formin2---input-fmnl2.tif]

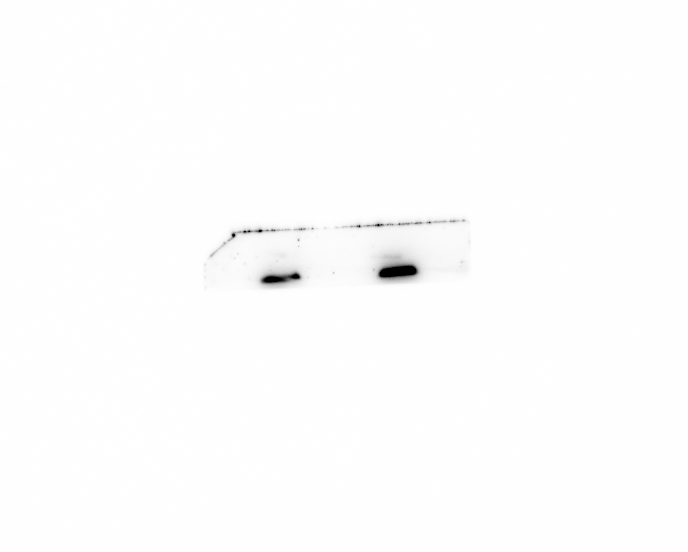

Supplement: Figure 4—source data 1. [file elife-92732-fig4-data1.zip › Fig 4/H---profilin---input-fmnl2-input-fmnl2.tif]

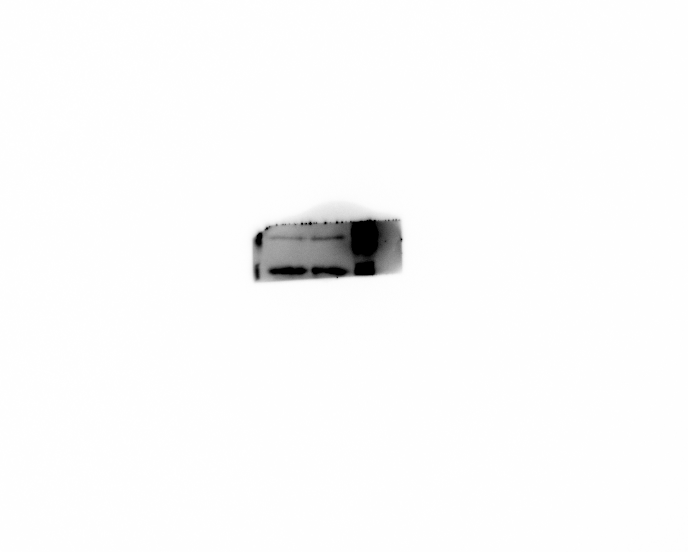

Supplement: Figure 4—source data 1. [file elife-92732-fig4-data1.zip › Fig 4/I---Arp2---C-fmnl2KD.tif]

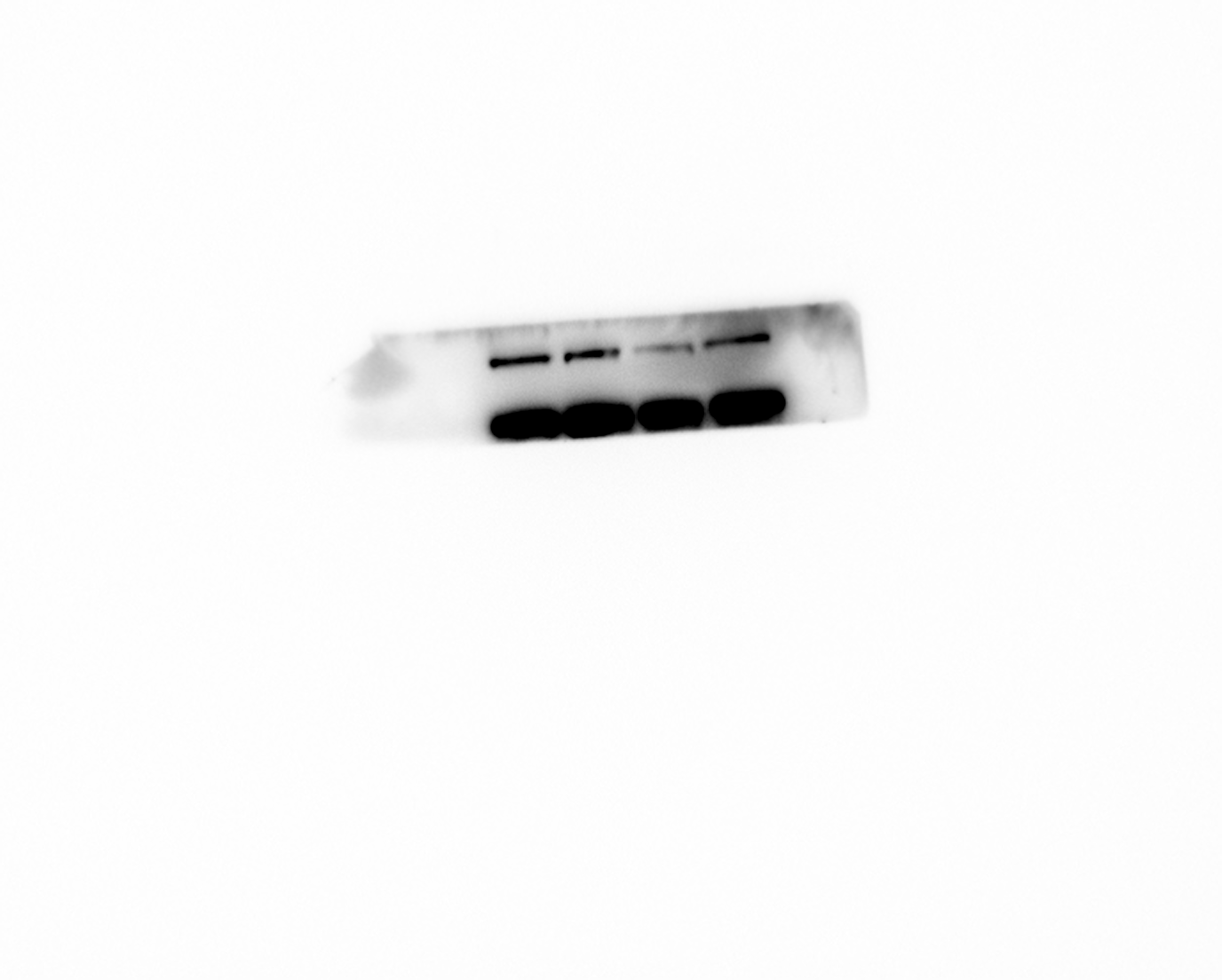

Supplement: Figure 4—source data 1. [file elife-92732-fig4-data1.zip › Fig 4/I---Arp2---fmnl2KD-fmnl2KD-200-400.tif]

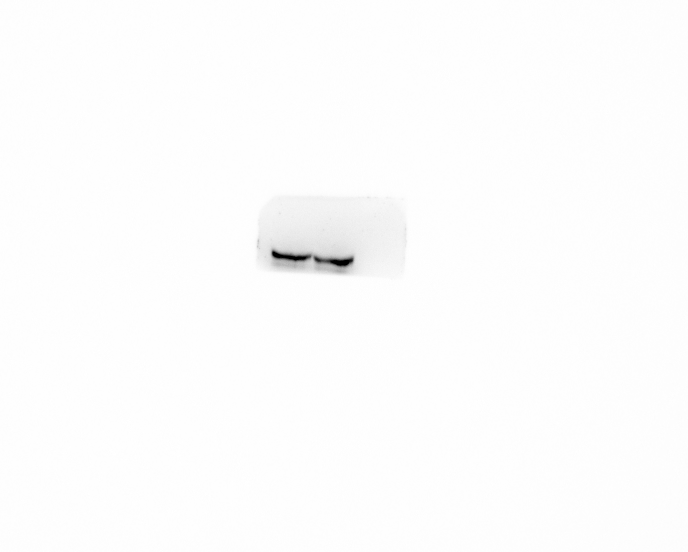

Supplement: Figure 4—source data 1. [file elife-92732-fig4-data1.zip › Fig 4/I---tubulin---C-fmnl2KD.tif]

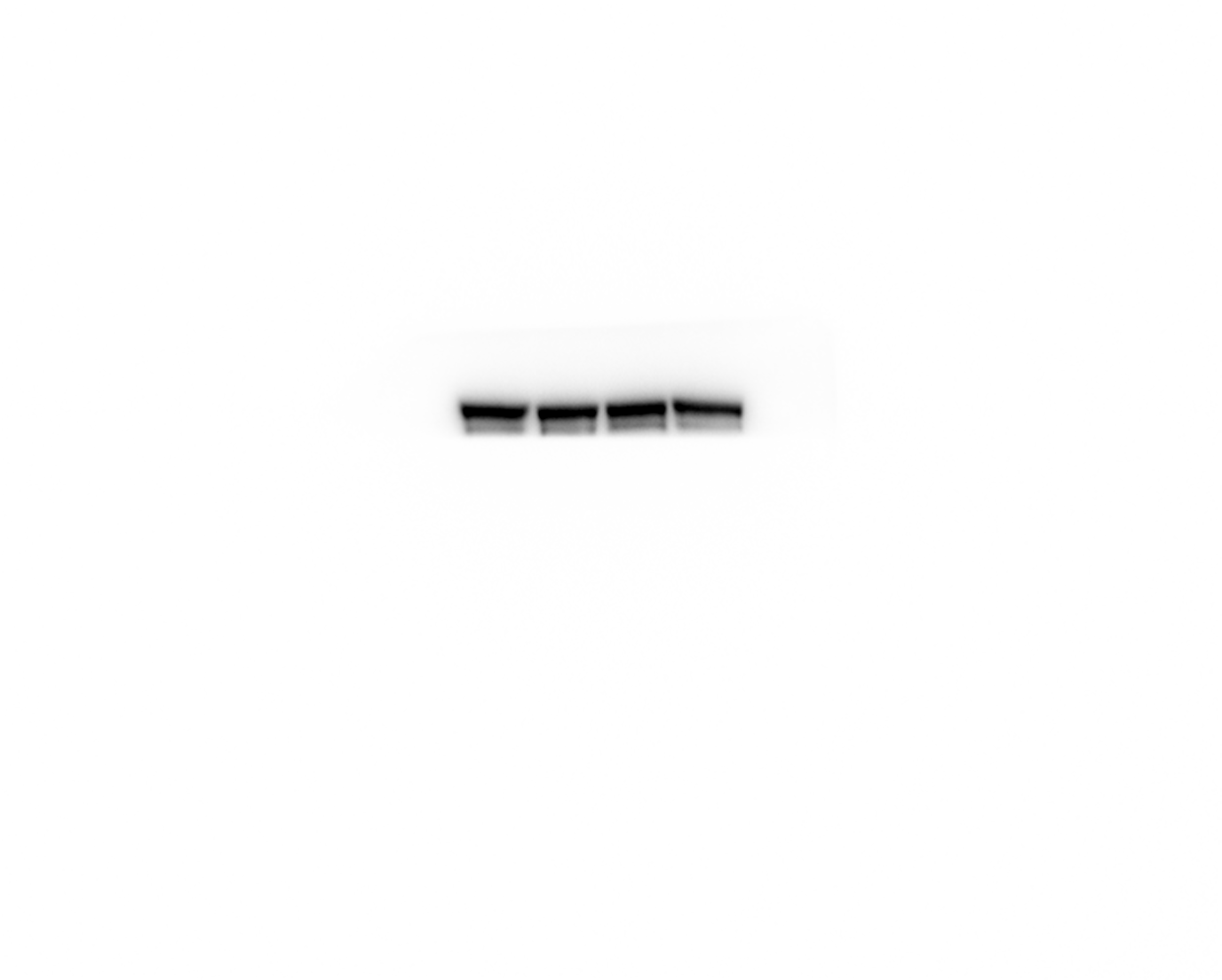

Supplement: Figure 4—source data 1. [file elife-92732-fig4-data1.zip › Fig 4/I---tubulin---fmnl2KD-fmnl2KD-200-400.tif]

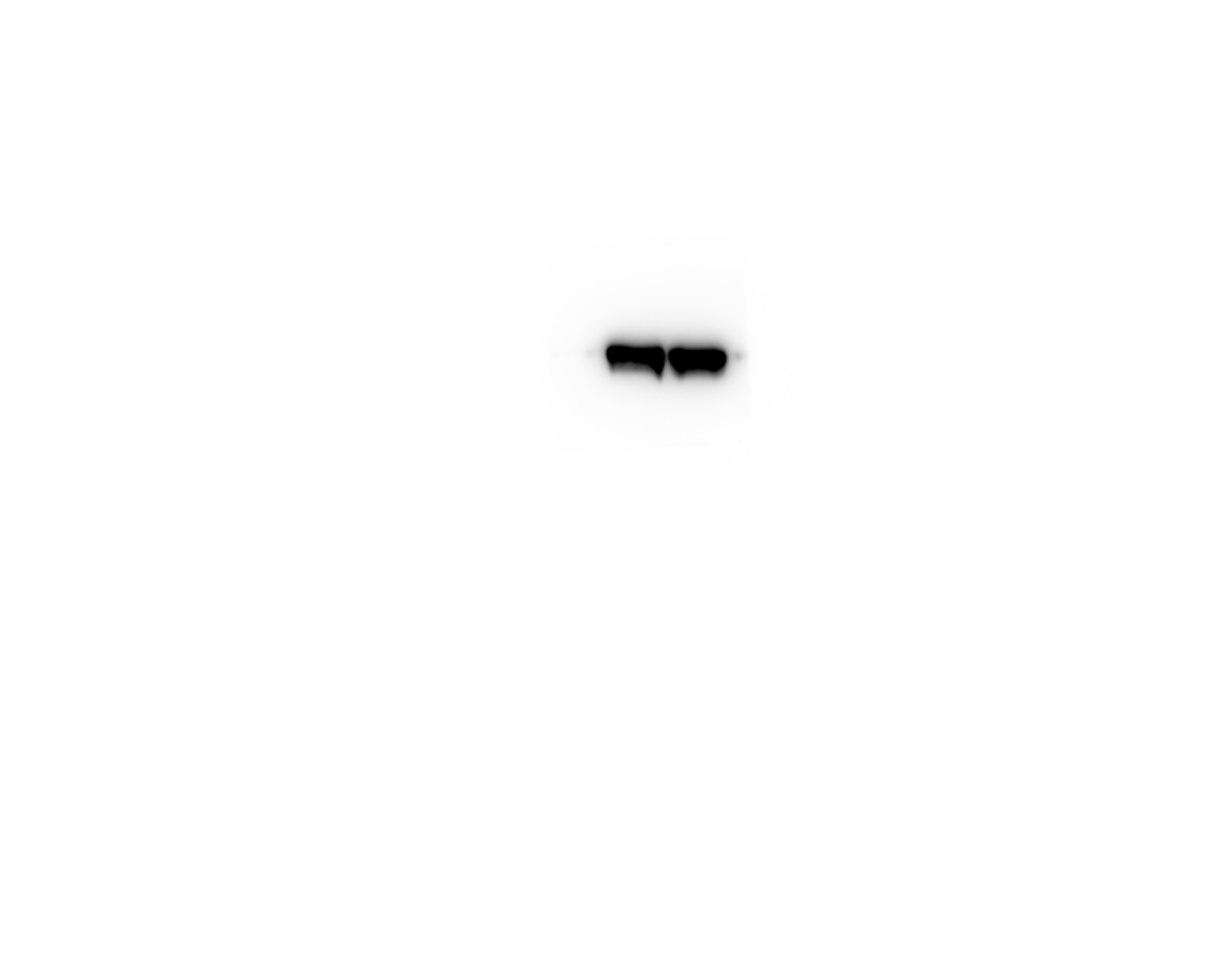

Supplement: Figure 4—source data 1. [file elife-92732-fig4-data1.zip › Fig 4/J---actin---fmnl2KD-200.tif]

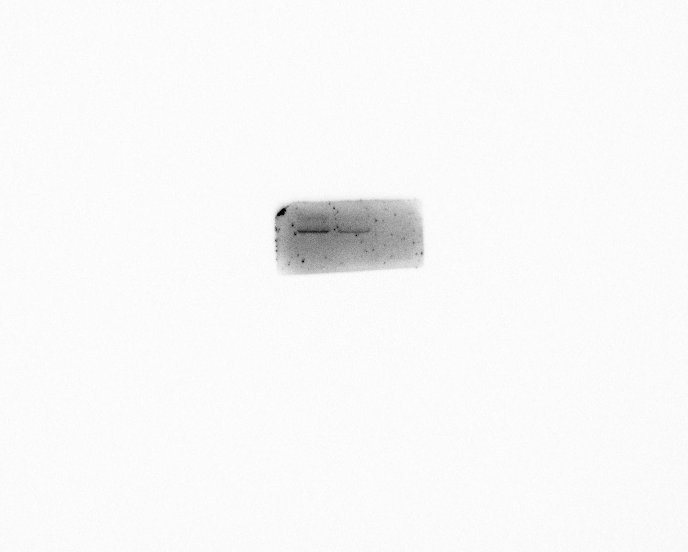

Supplement: Figure 4—source data 1. [file elife-92732-fig4-data1.zip › Fig 4/J---fmn2---C-fmnl2KD.tif]

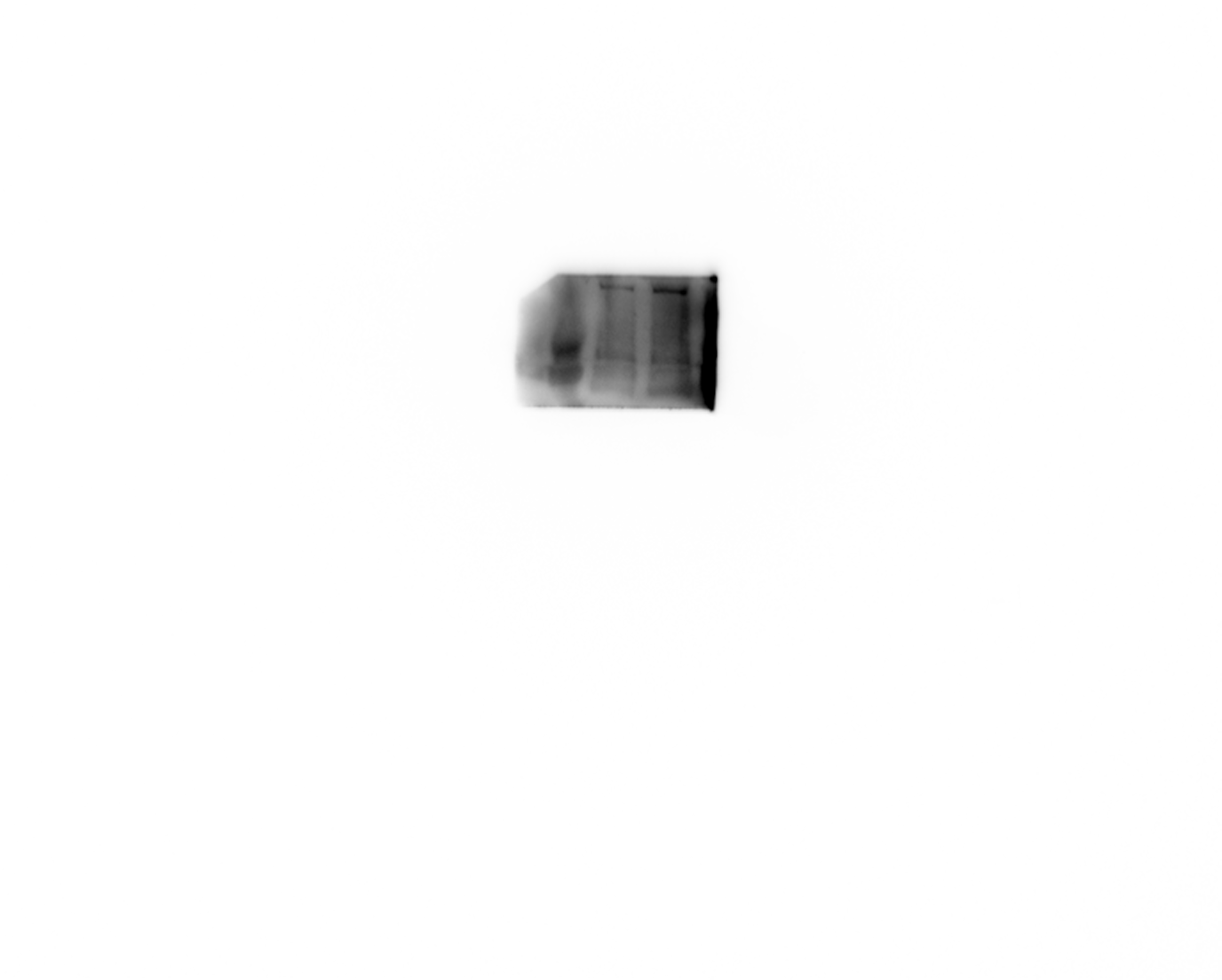

Supplement: Figure 4—source data 1. [file elife-92732-fig4-data1.zip › Fig 4/J---fmn2---fmnl2KD-200.tif]

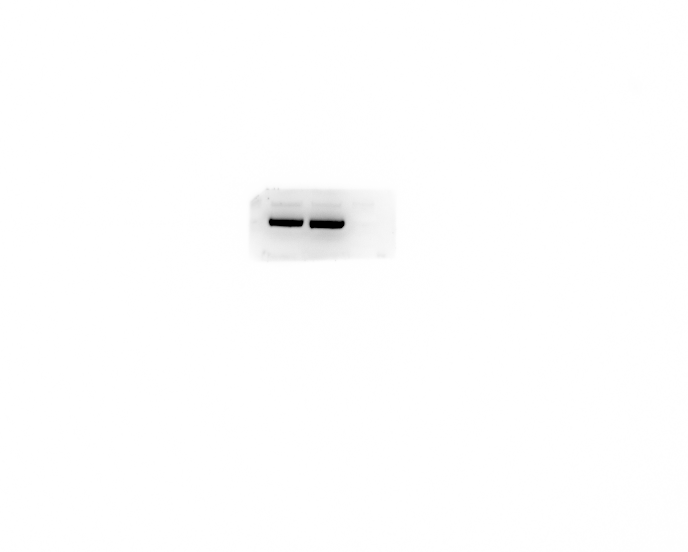

Supplement: Figure 4—source data 1. [file elife-92732-fig4-data1.zip › Fig 4/J---tubulin---C-fmnl2KD.tif]

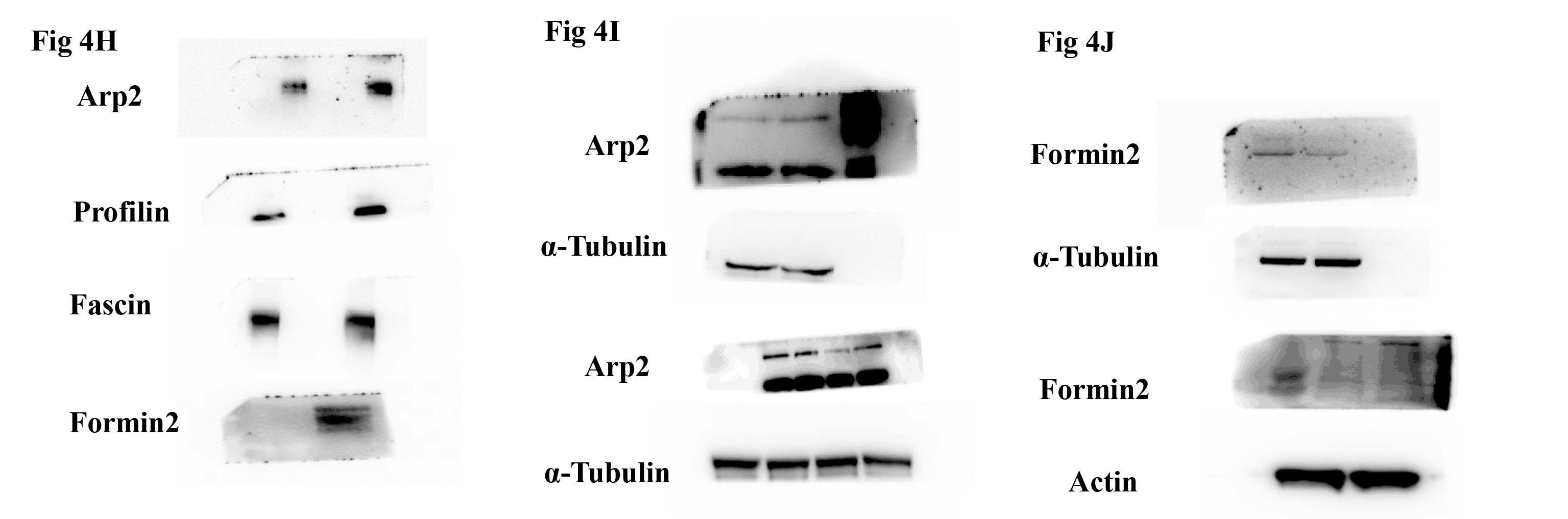

Supplement: Figure 4—source data 2. [file elife-92732-fig4-data2.zip › Figure 4 source data 2.tif]

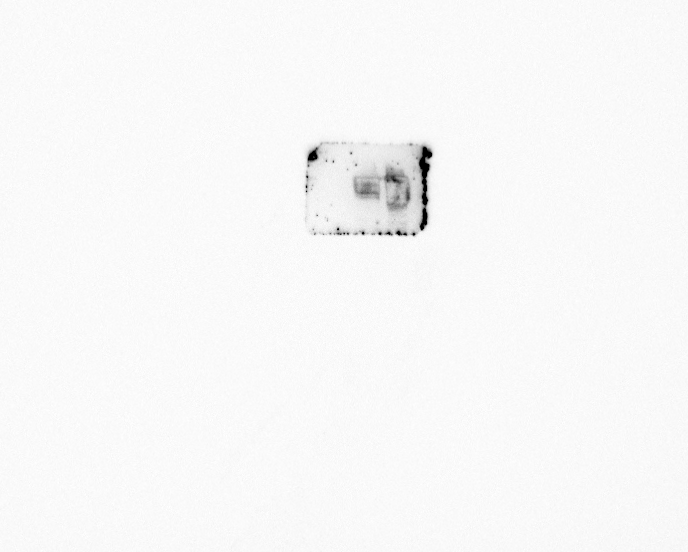

Supplement: Figure 5—source data 1. [file elife-92732-fig5-data1.zip › Fig 5/B---fmnl2---input-fmnl2.tif]

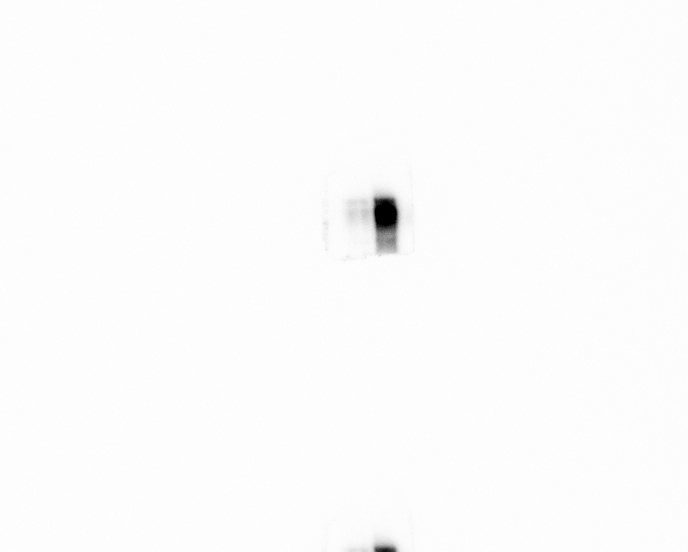

Supplement: Figure 5—source data 1. [file elife-92732-fig5-data1.zip › Fig 5/B---fmnl2---input-INF2.tif]

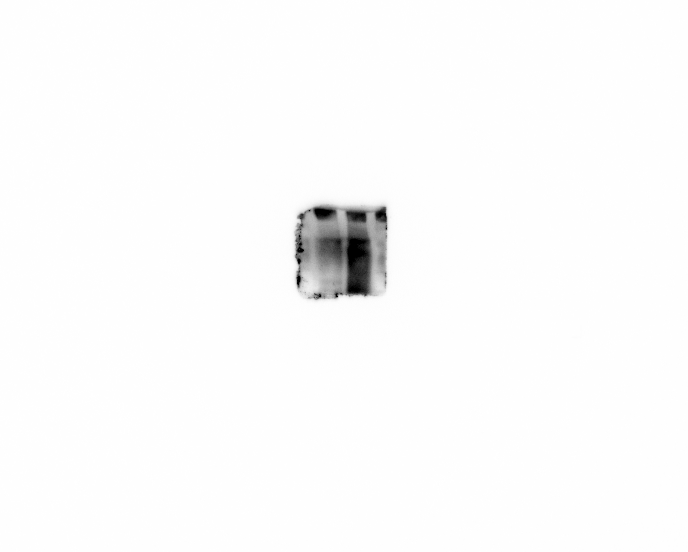

Supplement: Figure 5—source data 1. [file elife-92732-fig5-data1.zip › Fig 5/B---INF2---inpu-INF2.tif]

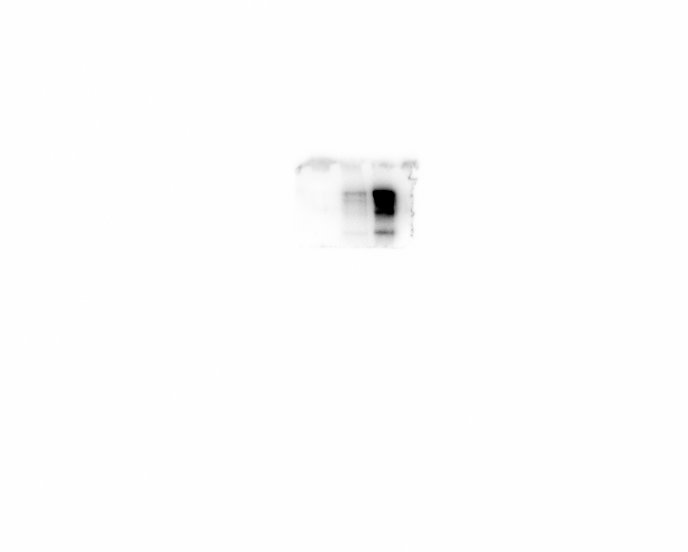

Supplement: Figure 5—source data 1. [file elife-92732-fig5-data1.zip › Fig 5/B---INF2---input-fmnl2.tif]

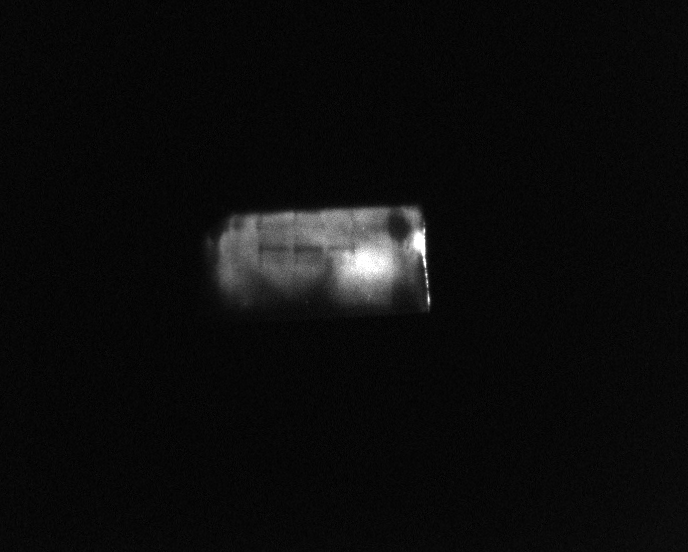

Supplement: Figure 5—source data 1. [file elife-92732-fig5-data1.zip › Fig 5/E---chop----C-fmnl2KD-fmnl2KD.tif]

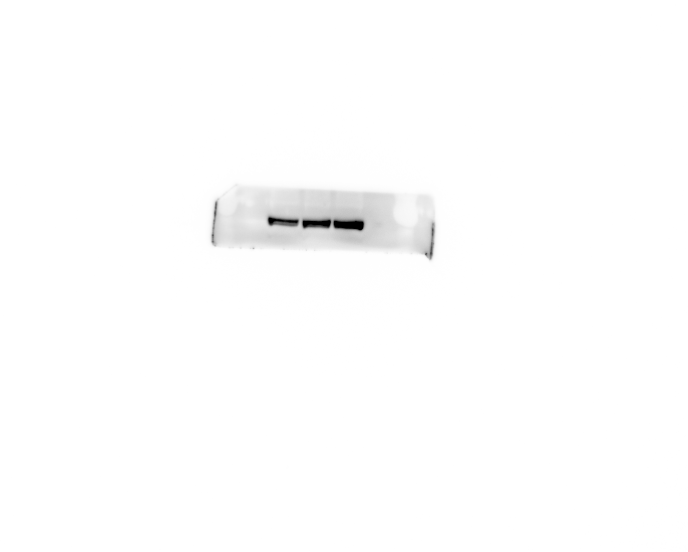

Supplement: Figure 5—source data 1. [file elife-92732-fig5-data1.zip › Fig 5/E---Grp78---C-fmnl2KD-fmnl2KD.tif]

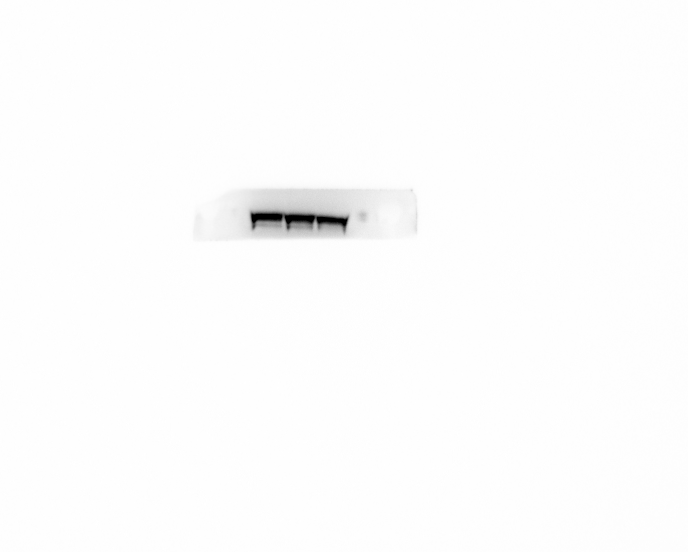

Supplement: Figure 5—source data 1. [file elife-92732-fig5-data1.zip › Fig 5/E---tubulin---C-fmnl2KD-fmnl2KD.tif]

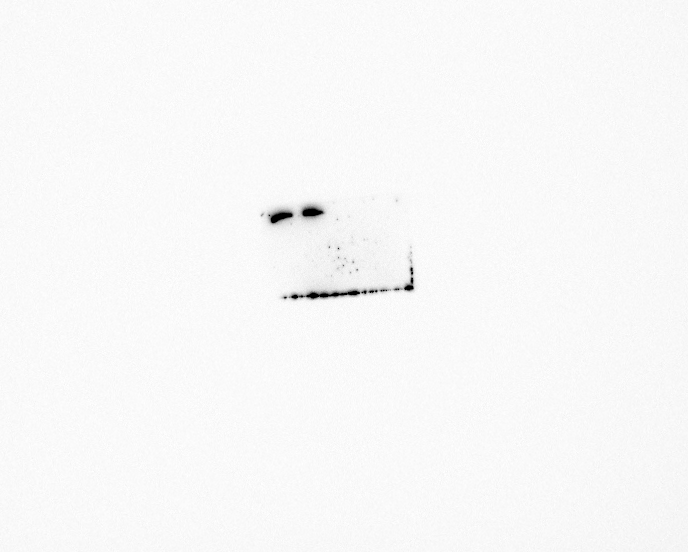

Supplement: Figure 5—source data 1. [file elife-92732-fig5-data1.zip › Fig 5/H---actin---fmnl2KD-rescue.tif]

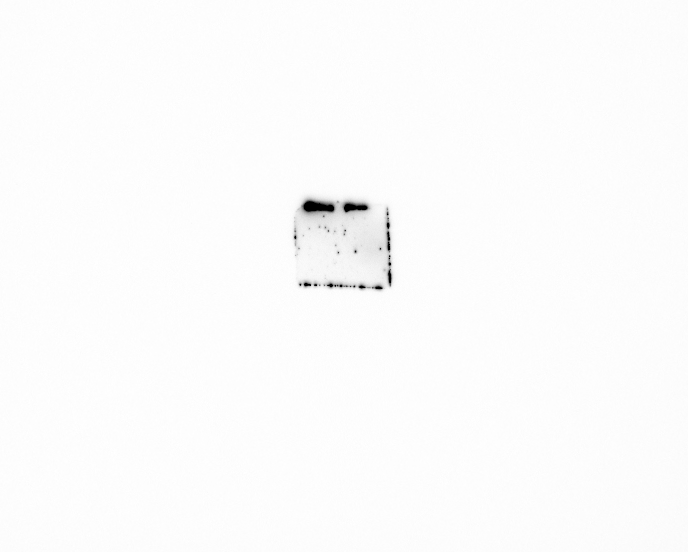

Supplement: Figure 5—source data 1. [file elife-92732-fig5-data1.zip › Fig 5/H---Grp78---fmnl2KD-rescue.tif]

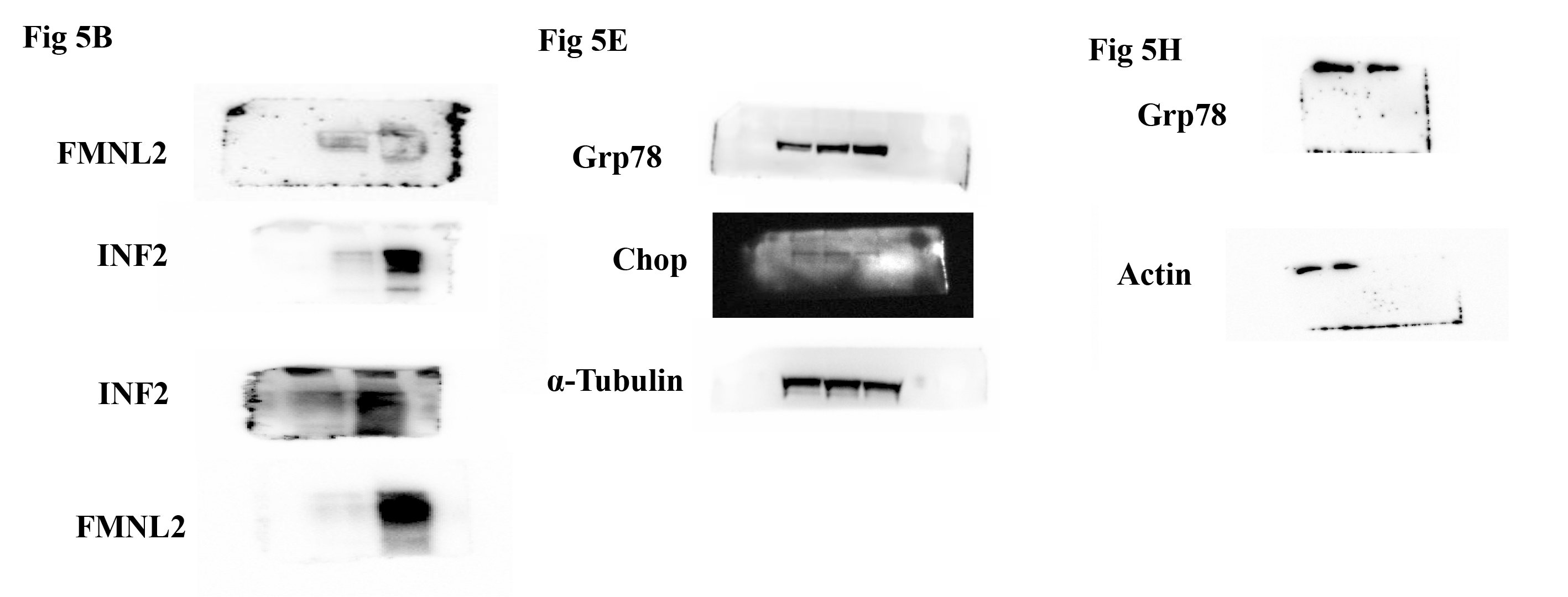

Supplement: Figure 5—source data 2. [file elife-92732-fig5-data2.zip › Figure 5 source data 2.tif]

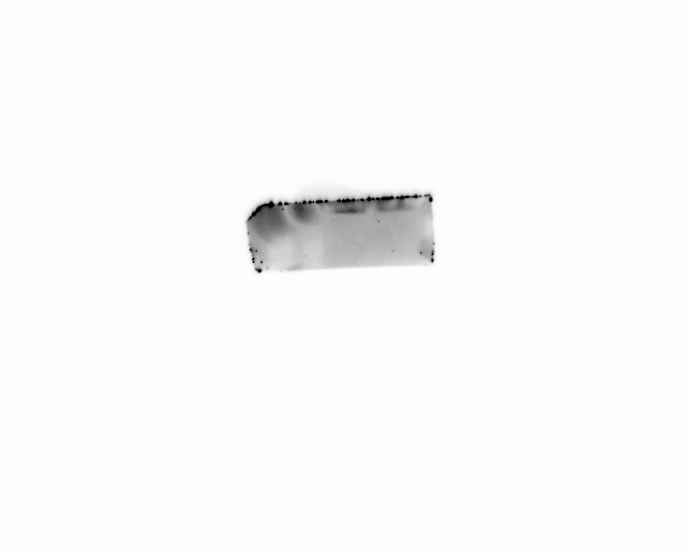

Supplement: Figure 6—source data 1. [file elife-92732-fig6-data1.zip › Fig 6/COFILIN---c-fmnl2KD-5.tif]

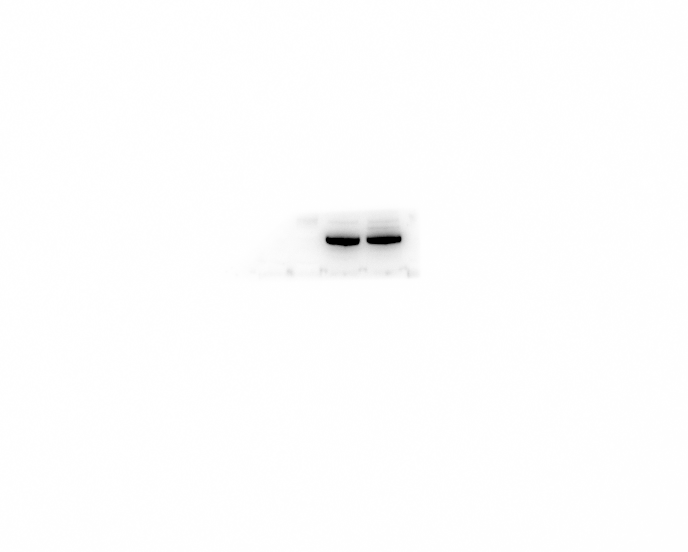

Supplement: Figure 6—source data 1. [file elife-92732-fig6-data1.zip › Fig 6/tubulin-c-fmnl2kd.tif]

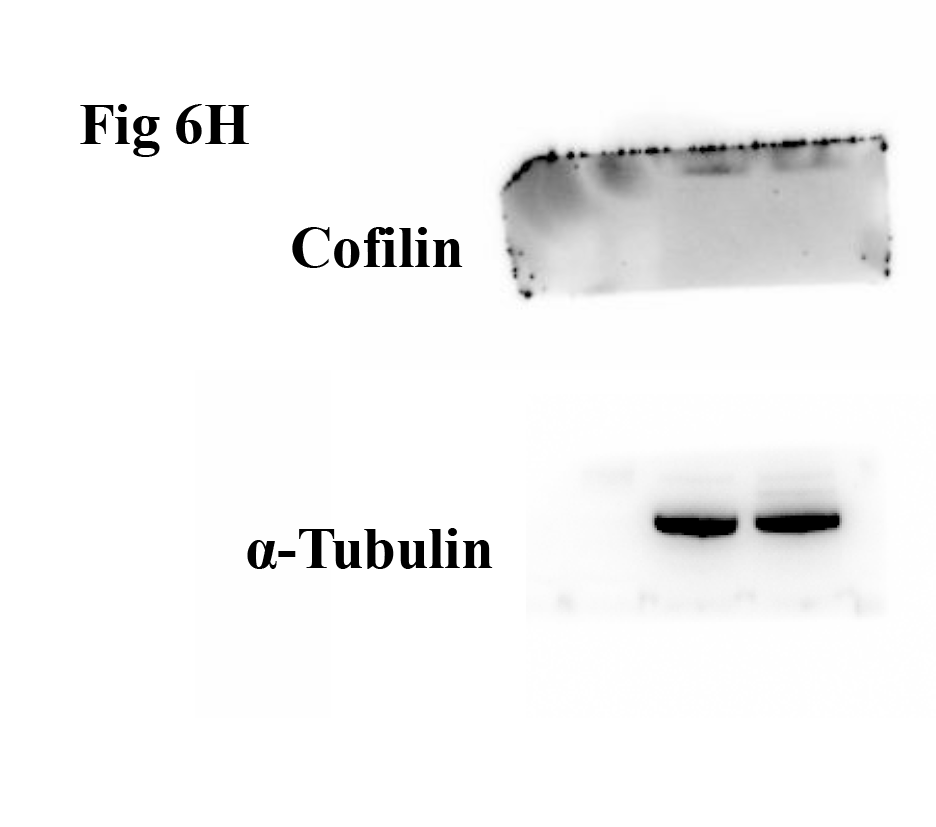

Supplement: Figure 6—source data 2. [file elife-92732-fig6-data2.zip › Figure 6 source data 2.tif]
